# Trends and adverse pregnancy outcomes associated with post-traumatic stress disorder: a population-based study of delivery hospitalisations

**DOI:** 10.1007/s00404-026-08448-6

**Published:** 2026-05-02

**Authors:** Dervla Quinn, Michael Donnelly, Joseph P. M. Kane, Lisa Kent, Ciaran O’Neill

**Affiliations:** Centre for Public Health (CPH), Queen’s University Belfast, Belfast, UK

**Keywords:** Post-traumatic stress disorder, Prevalence, Adverse pregnancy outcomes, Healthcare utilisation, Healthcare Cost and Utilization Project National Inpatient Sample

## Abstract

**Purpose:**

This study examined the prevalence of post-traumatic stress disorder (PTSD) diagnoses among pregnant women who delivered in hospitals in the United States between 2016 and 2020, and explored associations with adverse pregnancy outcomes, hospital length of stay, and hospital costs.

**Methods:**

This cross-sectional study utilised survey-weighted data from the Healthcare Cost and Utilization Project (HCUP) Nationwide Inpatient Sample (NIS) to estimate sample characteristics and prevalence trends. Logistic regression models were used to analyse associations between PTSD and adverse pregnancy outcomes. Length of hospital stay and hospital costs were examined using negative binomial and generalised linear models with log link and gamma distribution, respectively.

**Results:**

PTSD prevalence increased from 236.3 to 545.8 per 100,000 delivery hospitalisations between 2016 and 2020 (p < 0.001; average annual percentage change [AAPC] 23.0%). PTSD was associated with a higher prevalence of comorbidity, increased odds of preterm delivery (adjusted odds ratio [aOR] 1.13; 95% CI 1.08–1.18), and increased odds of fetal growth restriction (aOR 1.09; 95% CI 1.01–1.17, p = 0.03). Longer hospital stays and higher costs were also observed among women with PTSD.

**Conclusion:**

These findings highlight a rising prevalence of PTSD among pregnant women who delivered in hospitals in the United States over the study period. PTSD was associated with higher prevalence of comorbidity, and increased length of stay and hospital cost. Further research is warranted to investigate the reasons behind the trend, and to clarify the temporal relationship between prenatal PTSD and adverse pregnancy outcomes.

**Supplementary Information:**

The online version contains supplementary material available at 10.1007/s00404-026-08448-6.

## "What does this study add to the clinical work

**Table:** 

This study highlights an increasing prevalence of post-traumatic stress disorder (PTSD) among women who delivered in the United States over the study period, pointing towards the importance of increasing awareness among healthcare professionals involved in perinatal care. We observed a higher prevalence of comorbidity among women with PTSD, and examined associations with adverse pregnancy outcomes, hospital length of stay, and hospital cost."	

## Introduction

Post-traumatic stress disorder (PTSD) is characterised by the persistence of intense reactions to reminders of a traumatic event, avoidance, alteration of mood and cognition, a sense of imminent threat, hypervigilance, and insomnia following a traumatic event, with symptoms present for more than a month [1]. A systematic review estimated the prevalence of PTSD to be 3.3% during pregnancy in community samples, and 4.0% after birth [2]. The prevalence in high-risk populations in the same systematic review was estimated to be 19.0% before delivery, and 18.5% after delivery (high-risk populations were defined as women with difficult or traumatic births, severe fear of birth, severe pregnancy complications, or a history of abuse).

Evidence suggests that PTSD prevalence during pregnancy may be increasing. In a study investigating the association between severe maternal morbidity and PTSD, Duval et al. [3] reported an increase in PTSD in delivery hospitalisations in the United States between 2016 and 2019. However, the exclusion of women with other mental health conditions and substance use disorders limits the generalisability of these findings to the overall population.

The relationship between PTSD and maternal and pregnancy outcomes is not clearly established. A recent meta-analysis reported an association between PTSD and adverse pregnancy outcomes, including preterm birth and low birth weight (LBW), but noted that the overall evidence was mixed and considered to be of low quality [4]. An earlier meta-analysis similarly identified increased risk of preterm birth and LBW in women with prenatal PTSD [5].

This study aims to characterise the prevalence of PTSD diagnoses during delivery hospitalisations in the United States between 2016 and 2020, summarise maternal characteristics, and explore the association with adverse pregnancy outcomes, length of hospital stay, and hospital costs. To achieve this, we utilised data from the Healthcare Cost and Utilization Project (HCUP) Nationwide Inpatient Sample (NIS) using a cross-sectional study design to identify and compare delivery hospitalisations among women with and without PTSD.

## Methods

### Data source

This study used data from the HCUP NIS between 2016 and 2020. The HCUP NIS database is a 20% stratified sample of all discharges from United States hospitals, covering more than 97% of the United States population and is provided with discharge weights to enable calculation of nationwide estimates [6].

The database is derived from administrative billing data from hospital discharges, including Medicaid funded-hospitalisations, and contains clinical information coded using International Classification of Diseases, Clinical Modification, (ICD-CM) codes. To ensure coding consistency following the transition from ICD-9-CM to ICD-10-CM in 2015, analyses were limited to data from 2016 onwards.

### Study population

Hospital discharge records of deliveries between 2016 and 2020 were identified from the HCUP NIS by previously described methods [7, 8]. The unit of analysis in the HCUP NIS is the hospital discharge and given that hospitalisations for two separate deliveries within a single year are uncommon, each discharge record was treated as a separate delivery. Consistent with previous studies [9, 10], discharge records are referred to as individuals, to aid interpretation. The study population was restricted to females aged 12–55 years.

### Study variables

ICD-10-CM codes used to identify PTSD are provided in the supplementary information. To reduce the likelihood that the diagnosis was directly related to the delivery hospitalisation the ICD-10-CM code for acute PTSD (F43.11) was not included (accounting for < 0.5% of all deliveries with a PTSD diagnosis).

Maternal age was categorised into five-year intervals, with individuals aged < 20 years and 40 years or older assigned to distinct categories. Other characteristics provided by HCUP included race/ethnicity, payer, and estimated median household income of residents in the mother’s ZIP code (quartiles 1–4, with 4 representing the highest income quartile). Hospital characteristics included teaching status and location (rural, urban teaching, or urban nonteaching), and broad geographical region (Northeast, Midwest, South, and West).

ICD-10-CM codes for adverse pregnancy outcomes (preterm delivery and fetal growth restriction), and maternal characteristics (hypertension, diabetes, obesity, tobacco use, alcohol related disorders, other mental health conditions [depressive disorders, bipolar and related disorders, schizophrenia spectrum disorder], and other substance use disorders) are provided in the supplementary information. Codes specific to gestational diabetes and gestational hypertension were not included.

The Elixhauser Comorbidity Index, a validated algorithm that is recommended for use to measure comorbid conditions [11], was categorised to 0, 1, 2, and ≥ 3 comorbidities (similar to Brown et al. [12]).

Hospital specific cost-to-charge ratios provided by HCUP were applied, with costs adjusted to 2020 United States dollars using the Personal Consumption Expenditures Hospital Care Index [13].

### Statistical analysis

#### Analytic approach

Survey weights were applied to produce nationally representative estimates. Study sample characteristics were summarised using weighted estimates, with chi-square tests to compare categorical variables. Survey-weighted quarterly and annual rates per 100,000 deliveries were calculated. Quarterly trends were estimated using linear regression of log-transformed prevalence rates (to stabilise the variance), with Newey-West robust standard errors to account for serial correlation. The Average Annual Percent Change (AAPC) was calculated.

Multivariable survey-weighted logistic regression models examined associations between PTSD and pregnancy outcomes. Results are presented as unadjusted and adjusted odds ratios (aOR) with 95% confidence intervals (CI). Model 1 adjusted for sociodemographic characteristics (age category, race and ethnicity, median household income quartile by ZIP code), hospital factors (primary expected payer, and location/teaching status of the hospital), and tobacco use. Model 2 included all Model 1 covariates plus the Elixhauser Comorbidity Index as a categorical variable to explore the impact of clinical comorbidity (0, 1, 2, and ≥ 3 comorbidity measures).

A sensitivity analysis restricted the sample to delivery stays below the 90th centile (i.e. ≤ 5 days for caesarean section deliveries and ≤ 3 days for other deliveries) to reduce the likelihood that the diagnosis of PTSD was associated with delivery-related events resulting in prolonged hospitalisation.

Length of hospital stay (LOS) was analysed using negative binomial regression to account for overdispersion. Hospital costs were analysed using a complete case approach with generalised linear models specifying a gamma distribution and log link, consistent with the skewed non-negative distribution of cost data. Exponentiated coefficients were interpreted as unadjusted and adjusted LOS ratios and cost ratios, with estimated adjusted mean LOS and mean cost. Models adjusted for sociodemographic variables (age category, race and ethnicity, median household income quartile by ZIP code), hospital factors (primary expected payer, and location/teaching status of the hospital, region), year, and tobacco use (Model 1), with additional adjustment for the Elixhauser Comorbidity Index in Model 2 as a categorical variable (0, 1, 2, and ≥ 3 comorbidity measures).

Records missing ethnicity, payer, and income quartile were coded as a separate category. Missing costs (< 1% of hospitalisation records) were imputed using the mean cost per Diagnosis Related Group (DRG) per year in a sensitivity analysis (supplementary information).

Reporting followed the Strengthening the Reporting of Observational Studies in Epidemiology (STROBE) guidelines [14]. Analyses were conducted in Stata 18 (StataCorp LLC), using the “svy” command suite to account for the complex survey design.

## Results

The sample included 3,631,910 delivery hospital discharge records, after applying sample weights this corresponded to an estimated 18,159,540 (95% CI 17,877,732 – 18,441,348) deliveries nationwide from 2016 to 2020.

### Sample characteristics

The survey-weighted total number of delivery hospitalisations with PTSD between 2016 and 2020 was 66,490 (95% CI 64,339 – 68,641), or 366.1 per 100,000 deliveries. As seen in Table 1, women with PTSD were more likely to be of White ethnicity (PTSD: 63.4% vs. No PTSD: 50.3%) or Native American ethnicity (1.2% vs. 0.7%), and more likely to reside in areas with the lowest median household income quartile (31.5% vs. 27.7%), compared with those without PTSD.

**Table 1:** Maternal characteristics of delivery hospitalisations with and without PTSD (survey-weighted estimates)

	No PTSD	PTSD	P value
N	18,093,050 (99.6%)	66,490 (0.4%)	
Age in years at admission	29.0 (5.8)	28.0 (6.1)	
*Race and ethnicity*			
White	9,108,654 (50.3%)	42,165 (63.4%)	< 0.001
Black	2,615,360 (14.5%)	10,135 (15.2%)	
Hispanic	3,625,594 (20.0%)	8195 (12.3%)	
Asian or Pacific Islander	1,080,169 (6.0%)	940 (1.4%)	
Native American	128,050 (0.7%)	795 (1.2%)	
Other	795,470 (4.4%)	1810 (2.7%)	
Missing	739,754 (4.1%)	2450 (3.7%)	
*Obesity*			
Not obese	16,049,201 (88.7%)	52,455 (78.9%)	< 0.001
Obesity	2,043,849 (11.3%)	14,035 (21.1%)	
*Hypertension*			
No hypertension	17,737,740 (98.0%)	63,470 (95.5%)	< 0.001
Hypertension	355,310 (2.0%)	3020 (4.5%)	
*Diabetes*			
No diabetes	17,891,490 (98.9%)	64,685 (97.3%)	< 0.001
Diabetes	201,560 (1.1%)	1805 (2.7%)	
*Depression*			
No depression	17,456,681 (96.5%)	41,160 (61.9%)	< 0.001
Depression	636,370 (3.5%)	25,330 (38.1%)	
*Bipolar and related disorders*			
No bipolar	17,957,275 (99.2%)	52,435 (78.9%)	< 0.001
Bipolar disorder	135,775 (0.8%)	14,055 (21.1%)	
*Schizophrenia spectrum disorder*			
No schizophrenia spectrum disorder	18,074,255 (99.9%)	64,350 (96.8%)	< 0.001
Schizophrenia spectrum disorder	18,795 (0.1%)	2140 (3.2%)	
*Tobacco use*			
No tobacco use	17,181,966 (95.0%)	48,800 (73.4%)	< 0.001
Tobacco use	911,084 (5.0%)	17,690 (26.6%)	
*Opioid use*			
No opioid use	17,958,615 (99.3%)	61,350 (92.3%)	< 0.001
Opioid use	134,435 (0.7%)	5140 (7.7%)	
*Cocaine use*			
No cocaine use	18,060,710 (99.8%)	65,025 (97.8%)	< 0.001
Cocaine use	32,340 (0.2%)	1465 (2.2%)	
*Cannabis use*			
No cannabis use	17,831,455 (98.6%)	58,985 (88.7%)	< 0.001
Cannabis use	261,595 (1.4%)	7505 (11.3%)	
*Alcohol related disorder*			
No alcohol related disorder	18,068,095 (99.9%)	65,250 (98.1%)	< 0.001
Alcohol related disorder	24,955 (0.1%)	1240 (1.9%)	
*Elixhauser with categories*			
0	10,611,443 (58.6%)	8585 (12.9%)	< 0.001
1	4,156,288 (23.0%)	17,170 (25.8%)	
2	2,173,114 (12.0%)	16,425 (24.7%)	
3 or more	1,152,205 (6.4%)	24,310 (36.6%)	
*Median household income national quartile for patient ZIP Code*			
1	5,012,913 (27.7%)	20,955 (31.5%)	< 0.001
2	4,551,257 (25.2%)	19,340 (29.1%)	
3	4,422,977 (24.4%)	15,840 (23.8%)	
4	3,943,103 (21.8%)	9505 (14.3%)	
Missing	162,800 (0.9%)	850 (1.3%)	
*Primary expected payer*			
Medicare	123,090 (0.7%)	3340 (5.0%)	< 0.001
Medicaid	7,654,161 (42.3%)	41,555 (62.5%)	
Private insurance	9,333,790 (51.6%)	17,405 (26.2%)	
Self–pay	458,310 (2.5%)	985 (1.5%)	
Other	490,155 (2.7%)	3080 (4.6%)	
Missing/no charge^a^	33,545 (0.2%)	125 (0.2%)	

^a^combined due to low cell counts

Women with PTSD had a higher prevalence of comorbidities, with approximately double the prevalence of obesity, hypertension and diabetes, relative to women without PTSD. They also had over ten times the prevalence of comorbid mental health conditions, including depressive disorders (38.1% vs. 3.5%), 26 times the prevalence of bipolar and related disorders (21.1% vs. 0.8%), and 32 times the prevalence of schizophrenia spectrum disorder (3.2% vs. 0.1%). There was also a notably higher proportion of comorbidities, with 36.6% vs. 6.4% having three or more long-term conditions as quantified by the Elixhauser Comorbidity Index.

Substance use was higher among those with PTSD compared with those without, including tobacco use (26.6% vs. 5.0%), cannabis use (11.3% vs. 1.4%), opioid use (7.7% vs. 0.7%), cocaine use (2.2% vs. 0.2%), and alcohol related disorders (1.9% vs. 0.1%).

### Prevalence over time

The prevalence of PTSD more than doubled between 2016 and 2020, increasing from 236.3 (95% CI 218.6–255.4) per 100,000 delivery hospitalisations to 545.8 (95% CI 513.3–580.3) per 100,000 delivery hospitalisations (Table 2, p < 0.001). The Average Annual Percent Change (AAPC) was 22.97% per year (95% CI 21.53–24.43%) between 2016 and 2020.

**Table 2:** Annual survey-weighted number of deliveries in women with PTSD

Year	Estimated number of deliveries (95% CIs)	Prevalence per 100,000 deliveries (95% CIs)
2016	8,940 (8,213 – 9,667)	236.3 (218.6 – 255.4)
2017	10,700 (9,911 – 11,489)	288.9 (269.1 – 310.1)
2018	12,665 (11,754 – 13,576)	348.5 (325.6 – 373.0)
2019	15,335 (14,298 – 16,372)	428.0 (401.7 – 456.0)
2020	18,850 (17,596 – 20,104)	545.8 (513.3 – 580.3)

**Fig. 1:**
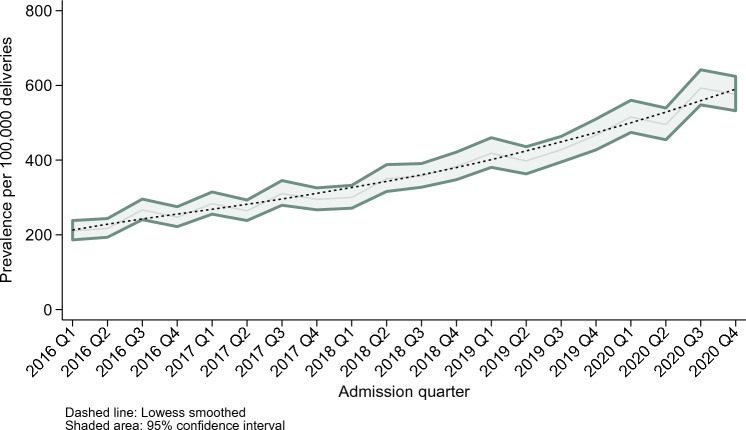
Survey-weighted quarterly prevalence of PTSD per 100,000 delivery hospitalisations from 2016 to 2020

Quarterly prevalence estimates per 100,000 delivery discharges are presented in Fig. 1.

### Association with adverse pregnancy outcomes

As can be seen in Table 3, women with PTSD had a higher proportion of adverse pregnancy outcomes compared with those without PTSD. Before adjustment, these women had a 90% increased odds of preterm delivery, and an approximately 65% higher odds of fetal growth restriction (FGR).

**Table 3:** Prevalence, unadjusted and adjusted results of association of PTSD and adverse pregnancy outcomes

Adverse pregnancy outcome	Women without PTSD (%)	Women with PTSD (%)	Unadjusted OR (95% CI)	Model 1 aOR (95% CI)	Model 2 aOR (95% CI)	P value (Model 2)
Preterm delivery	10.2%	17.8%	1.90 (1.81–1.99)^a^	1.55 (1.48–1.63)^a^	1.13 (1.08–1.18)	< 0.0001
FGR	3.5%	5.8%	1.66 (1.54–1.79)^a^	1.26 (1.17–1.36)^a^	1.09 (1.01–1.17)	0.0279

*OR* odds ratio, *aOR* adjusted odds ratio, *FGR* fetal growth restriction

^a^P value < 0.0001

Model 1: Adjusted for sociodemographic characteristics (age group, race and ethnicity, income), hospital factors (payer, hospital location), tobacco use

Model 2: Model 1 and Elixhauser clinical comorbidities index

**Fig. 2:**
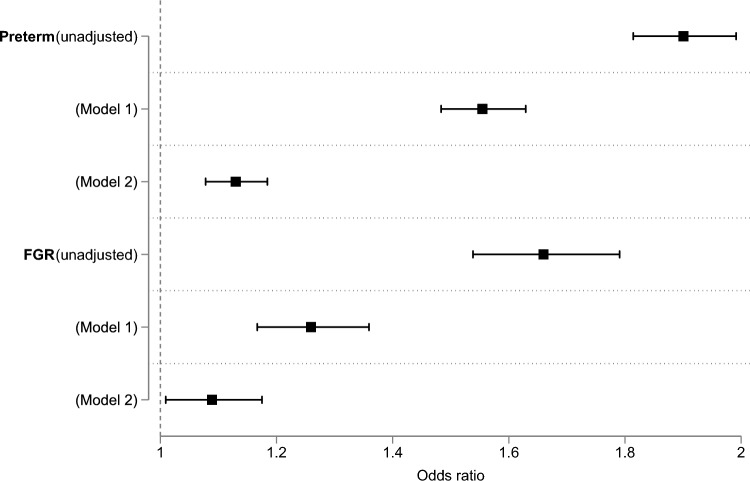
Unadjusted and adjusted odds ratios of adverse pregnancy outcomes in women with PTSD. FGR fetal growth restriction. Model 1: Adjusted for sociodemographic characteristics (age group, race and ethnicity, income), hospital factors (payer, hospital location), tobacco use. Model 2: Model 1 and Elixhauser clinical comorbidities index

When the results were adjusted, estimates were attenuated across the models relative to the unadjusted results. While statistically significant associations were observed for all outcomes in Model 1, in the fully adjusted model (Model 2), there was evidence of an association with preterm delivery, with 13% increased odds among women with PTSD (aOR 1.13; 95% CI 1.08—1.18), and less strong evidence of an association with fetal growth restriction (p = 0.03). The unadjusted and adjusted results are presented in Table 3 and Fig. 2.

Similar overall estimates were observed in the sensitivity analysis that excluded prolonged hospital stays (supplementary information), with the exception that the association with fetal growth restriction no longer reaching the conventional threshold for statistical significance (aOR 1.08; 95% CI 0.99–1.18, p = 0.08).

### Association with length of hospital stay and hospital costs

Delivery hospitalisations with PTSD had higher average length of stay (LOS) and hospital costs compared with those without the condition (Table 4). On average, LOS was 0.6 days longer, and hospital costs were on average $1,085 higher per delivery hospitalisation (based on median values).

**Table 4:** Length of stay (LOS) and costs by PTSD

	Women without PTSD	Women with PTSD
*Length of stay (days)*		
Mean (SD)	2.63 (2.25)	3.27 (3.72)
Median (IQR)	2 (2–3)	3 (2–3)
*Cost (US dollars)*		
Mean (SD)	5692 (4974)	7420 (8398)
Median (IQR)	4652 (3287–6702)	5737 (3984–8578)

After adjustment in the fully adjusted model (Model 2), PTSD was associated with an 8% increase in length of hospital stay (LOS) and a 6% increase in hospital costs (Table 5). These findings were consistent in a sensitivity analysis that imputed missing cost data (supplementary information).

**Table 5:** Unadjusted and adjusted length of stay (LOS) ratios and cost ratios of delivery hospitalisations among women with PTSD

Model	LOS ratio^a^ (95% CI)	Adjusted mean LOS, days (95% CI)	Cost ratio^b^ (95% CI)	Adjusted mean cost, USD (95% CI)
Unadjusted	1.25 (1.22–1.27)^c^	3.27 (3.21–3.34)	1.30 (1.27–1.33)^c^	7420 (7239–7602)
Model 1	1.23 (1.20–1.25)^c^	3.22 (3.16–3.29)	1.25 (1.22–1.27)^c^	7103 (6949–7256)
Model 2	1.08 (1.06–1.10)^c^	2.83 (2.77–2.88)	1.06 (1.04–1.08)^c^	6041 (5922–6159)

^a^Negative binomial regression

^b^Generalised linear model (GLM) with a gamma distribution and a log link function

^c^P value < 0.0001

Model 1: Adjusted for sociodemographic characteristics (age group, race and ethnicity, income), hospital factors (payer, hospital location, region), tobacco use, year

Model 2: Model 1 and Elixhauser clinical comorbidities index

## Discussion

PTSD diagnoses among delivery hospitalisations increased substantially during the study period, with PTSD more than doubling between 2016 and 2020, increasing from 236.3 to 545.8 per 100,000 delivery hospitalisations. The impact of the COVID-19 pandemic is unclear; however, restricting the analysis to 2016 to 2019 produced a similar AAPC of 21.77% (95% CI 21.41–22.13%), with visual inspection of the graph suggesting that the rate may have increased around 2018.

Duval et al. [3] found an association between severe maternal morbidity (SMM) and PTSD using data from the HCUP NIS, and reported an increase of approximately 75% between 2016 and 2019, from 50 per 100,000 deliveries to 88 per 100,000 deliveries. The prevalence in this study was higher; in the Duval et al. study [3], women with other mental health conditions (including depressive disorder, bipolar spectrum disorder, schizophrenia spectrum disorder, anxiety disorder, acute stress reaction, and adjustment disorder), and substance use disorders (including tobacco, alcohol, and drug use) were excluded (the reason provided was that these conditions are known to be associated with PTSD). Hall et al. [15] reported an almost 400% increase in perinatal PTSD prevalence (one year before and after delivery) between 2008 and 2020 in the US using a U.S. claims database, with an overall prevalence of 0.8%.

The rise in PTSD may reflect a true increase in prevalence, increased recognition and changes in clinical practice (including after the introduction of the Diagnostic and Statistical Manual of Mental Disorders, DSM-5, in 2013), increased awareness, and/or changes in administrative coding practices. Trends in other mental health conditions, have also been found to have increased using the same data source. For example, Weiss et al. [16] reported an increase in anxiety and obsessive–compulsive disorders in delivery hospitalisations of 74.3% in 2020 relative to 2017, and an increase of 61.3% in depressive disorders over the same period. Logue et al. [17] reported an increase of over 1,000% in the prevalence of mental health conditions (including depression, anxiety, bipolar spectrum disorder and schizophrenia spectrum disorder) in the delivery hospitalisations of women between the years 2000 and 2018 (increasing from 0.6 to 7.3%). Trends in cannabis and opioid use disorders in delivery hospitalisations between 2000 and 2018 have also been reported to have increased, with the increase observed across income quartiles, maternal age, and urban/rural hospital location [18].

The overall estimated prevalence of PTSD in delivery hospitalisations in the United States between 2016 and 2020 was 366.1 per 100,000 deliveries. This estimate is lower than the prevalence reported in the systematic review by Yildiz et al. [2], and likely reflects a number of factors, including the cross-sectional nature of HCUP NIS data (which are derived from administrative billing information in discharge records), as well as methodological differences across studies included in the systematic reviews (e.g., differences in case identification, differences in sampling techniques, different population characteristics, and variations in clinical practice). With respect to trends in other countries, hospitalisations with a diagnosis of PTSD doubled in general hospitals in France between 2013 and 2022, with a relatively higher prevalence observed among younger female patients [19].

Comorbid health conditions and behavioural health risk factors, including tobacco and other substance use, were more commonly observed in women with PTSD compared with those without PTSD. Women with a diagnosis of PTSD were approximately 6 times more likely to have ≥ 3 Elixhauser comorbidity measures compared to the overall sample (36.6% vs. 6.4%). This was particularly notable in relation to comorbid mental health conditions. This is consistent with other studies in non-obstetric populations, for example, a UK sample reported a self-reported prevalence of psychiatric comorbidity of almost 80% in individuals with PTSD, most commonly depression [20].

The results of this study suggest an association between PTSD and adverse pregnancy outcomes that was attenuated after adjustment for potential confounding factors, with the odds of adverse pregnancy outcomes (preterm delivery in particular) reduced after adjustment for clinical comorbidities, emphasising the impact of comorbidity. However, these results do not imply causation and should be interpreted with caution due to the cross-sectional nature of the data and the unavailability of a present-on-admission indicator. It is recommended that PTSD symptoms are present for more than one month before a diagnosis is made, reducing the likelihood that the PTSD observed was first diagnosed and directly related to the specific delivery [21]. Despite this, due to the nature of the data and unavailability of a present-on-admission indicator, it is not possible to determine with certainty whether the diagnosis of PTSD preceded the pregnancy, was first diagnosed during the pregnancy, or was first diagnosed during the delivery hospitalisation. Childbirth is increasingly recognised as a potentially traumatic event that may result in PTSD [22, 23], and this is particularly relevant for complicated deliveries; these results should therefore be interpreted with caution. To reduce the likelihood of identifying PTSD diagnoses related to the specific delivery, the ICD-10-CM code for acute PTSD was not included in the analysis (and accounted for less than 1% of all PTSD diagnoses in the sample), and a sensitivity analysis that excluded prolonged hospitalisations found similar findings with respect to preterm delivery, however the association with FGR no longer reached the threshold for statistical significance.

Using HCUP NIS data from 2017 only, Dubey et al. [26] examined the association between a number of mental health conditions, including PTSD, and various pregnancy outcomes in delivery hospitalisations, and reported an association between PTSD and preterm birth (aOR 1.40; 95% CI 1.19–1.64). Zitoun et al. [4] published a systematic review and meta-analysis investigating the association between PTSD and pregnancy outcomes, including 15 studies investigating LBW, and 14 studies investigating preterm birth [4]. Whilst noting that the overall findings from the studies for both of these outcomes were mixed, an increased risk was reported for both outcomes in women with PTSD when the results were pooled in a meta-analysis for both LBW (OR 2.05; 95% CI 1.27–3.33) and preterm birth (OR 1.23; 95% CI 1.11–1.37). The authors noted that the quality of the evidence was low. Similar findings were found in an earlier systematic review and meta-analysis by Sanjuan et al. [5] in women with prenatal PTSD, with an increased risk of LBW (OR 1.96; 95% CI 1.26–3.03) when the results from 10 studies were pooled, and an increased risk of preterm birth when the results from 11 studies were pooled (OR 1.42; 95% CI, 1.16–1.73). An earlier systematic review reported inconsistent evidence for an association between PTSD during pregnancy and fetal growth and preterm birth [27].

Proposed mechanisms linking stress-related disorders to adverse pregnancy outcomes include dysregulation of the hypothalamic–pituitary–adrenal axis and activation of pro-inflammatory pathways [24, 25]. In addition, there is a possibility of confounding factors that are not captured within these data, these include adversity and trauma (including adverse childhood experiences), not necessarily associated with PTSD. There is also a possibility of common genetic and familial causative factors.

Regarding the findings of higher hospital costs and length of stay, Brown et al. [28] analysed the impact of perinatal mental health conditions on hospital cost and length of stay using data from 2016–2017 from the HCUP NIS, and found the highest associated cost per delivery in those with “trauma/stress-related disorders” (including PTSD, adjustment disorder, and acute stress reactions).

### Strengths

This study has several strengths. The HCUP NIS is a large and nationally representative population-based dataset. Data from 2016 onwards were analysed to ensure consistency following the introduction of ICD-10-CM coding in 2015. Findings are therefore broadly generalisable to hospital births, which account for the vast majority of deliveries in the US. In 2017, only 1.6% of births occurred outside hospitals [29]. In addition, women with other mental health conditions were not excluded, allowing estimation of national trends in delivery hospitalisations in the overall population.

### Limitations

However, several limitations are acknowledged. Most importantly, given the cross-sectional design of this study, the temporal sequence of diagnoses cannot be established, and given the complex relationship between mental health conditions, comorbidities, and behavioural risk factors, it is not possible to determine causation based on these results but rather to observe associations. Information relating to how (including by whom and the criteria used) and when PTSD was first diagnosed is not available. Due to the lack of a present-on-admission indicator, this limitation relating to the temporal sequence of events is also relevant to the sensitivity analysis that excluded prolonged admissions (i.e. the possibility that PTSD was diagnosed during rather than before the delivery hospitalisation cannot be excluded).

Baseline differences were observed between women with PTSD, and those without, including large differences in the prevalence of psychiatric comorbidities such as depression, and the proportion with three or more Elixhauser comorbidity measures. Although multivariable logistic regression in Model 2 adjusted for the number of Elixhauser comorbidity measures as described (the Elixhauser comorbidity index includes measures relating to psychiatric comorbidity, drug abuse, and alcohol abuse), residual confounding cannot be excluded. Alternative analytical approaches, such as propensity score methods, could be considered in future analyses.

The HCUP NIS is derived from administrative billing data, which may underestimate the prevalence of comorbidities and mental health conditions, introducing potential misclassification bias [30]. In addition, it does not capture diagnosis severity, pharmacological treatment, parity or other aspects of prior obstetric history across pregnancies, and the cross-sectional design prevents longitudinal follow-up. Also, delivery records cannot be linked to neonatal outcomes or other pregnancies in the same individual. The increased likelihood of highly significant p values when analysing large datasets, and the need for caution when interpreting results, is also acknowledged [31]. Some covariates are only available at broader geographic level (e.g. household income is measured at ZIP code level). Finally, this study utilised data from the United States, and therefore the generalisability of the results to other populations is uncertain.

### Implications for practice and future research

Optimising the management of women with PTSD, including management of comorbidities (both physical and mental health) and behavioural risk factors, may help improve outcomes for both mothers and infants. The findings also suggest that these women may benefit from preconception care planning, with health services working to optimise management of long-term physical and mental health conditions before future pregnancies.

Future research is warranted to examine trends in more recent years in more detail, and also to further clarify the temporal relationship with pre-existing prenatal PTSD and maternal and infant outcomes.

## Conclusion

In this study, PTSD during delivery hospitalisations was increasingly prevalent over the study period and was associated with higher prevalence of comorbid psychiatric and medical conditions, increased prevalence of behavioural risk factors (including tobacco and other substance use), increased odds of adverse pregnancy outcomes, longer hospital stays, and higher hospital costs.

## Supplementary Information

Below is the link to the electronic supplementary material.

Supplementary file1 (DOCX 50 KB)

## Data Availability

This study used the Healthcare Cost and Utilization Project (HCUP) Nationwide Inpatient Sample (NIS) data from 2016 to 2020 administered by the Agency for Healthcare Research and Quality (AHRQ) and available at https://hcup-us.ahrq.gov.
