# Supplementary material for: Trends and adverse pregnancy outcomes associated with post-traumatic stress disorder: a population-based study of delivery hospitalisations
Source: Arch Gynecol Obstet. 2026 May 2;313(1):191. doi: 10.1007/s00404-026-08448-6 (PMC13135547; doi:10.1007/s00404-026-08448-6)
Supplement: Supplementary file 1 — Supplementary file1 (DOCX 50 KB) [file 404_2026_8448_MOESM1_ESM.docx]

**Supplementary information**

**Appendix 1: Code lists**

| **Deliveries** | ICD-10-CM inclusion codes | Z37* O80* O82* O7582 |
| --- | --- | --- |
|  | ICD-10-PCS inclusion codes | 10D00Z0 10D00Z1 10D00Z2 10D07Z3 10D07Z4 10D07Z5 10D07Z6 10D07Z7 10D07Z8 10E0XZZ |
|  | MS-DRG inclusion codes | 765, 766, 767, 774, 775, 768, 783, 784, 785, 786, 787, 788, 796, 797, 798, 805, 806, 807 |
|  | ICD-10-CM exclusion codes | O00* O01* O02* O03* O04* O07* O08* |
|  | ICD-10-PCS exclusion codes | 10A0* |
| **Post-traumatic stress disorder (PTSD)** | | |
| PTSD | ICD-10-CM | F431 F4310 F4312 |
| **Other mental health disorders** | | |
| Depressive disorders | ICD-10-CM | F320 F321 F322 F323 F324 F325 F328 F329 F33* F341 |
| Bipolar and related disorders | ICD-10-CM | F30* F31* F340 |
| Schizophrenia spectrum disorder | ICD-10-CM | F20* F25* |
| **Outcomes** |  |  |
| Preterm delivery | ICD-10-CM | O601* Z3A2* Z3A30/Z3A36 O42012/O42019 |
| Fetal growth restriction | ICD-10-CM | O3659* |
| **Comorbidities** |  |  |
| Obesity | ICD-10-CM | E660/E662 E668/E669 O9921* Z683* Z684* |
| Diabetes | ICD-10-CM | E08* E09* E10* E11* E13* O240* O241* O243* |
| Hypertension | ICD-10-CM | I1* O10* |
| **Substance use disorders** |  |  |
| Opioid use | ICD-10-CM | F11* |
| Cannabis use | ICD-10-CM | F12* |
| Cocaine use | ICD-10-CM | F14* |
| Alcohol related disorders | ICD-10-CM | F101* F102* F10921/F1099 O354* O99310/O99314 |
| Tobacco use | ICD-10-CM | F172* O9933* |

**Sensitivity analysis – PTSD analysis excluding prolonged hospital stays**

Adjusted results of association of PTSD and adverse pregnancy outcomes (excluding prolonged hospital stays)

| **Adverse pregnancy outcome** | **Model 1 aOR (95% CI)** | **P value** | **Model 2 aOR (95% CI)** | **P value** |
| --- | --- | --- | --- | --- |
| Preterm delivery | 1.41 (1.34 - 1.49) | <0.0001 | 1.11 (1.05 – 1.17) | 0.0004 |
| FGR | 1.21 (1.11 - 1.32) | <0.0001 | 1.08 (0.99 – 1.18) | 0.0752 |

*Abbreviations*: aOR, adjusted odds ratio, FGR, intrauterine growth restriction.

Model 1: adjusted for sociodemographic characteristics (age group, race and ethnicity, income), hospital factors (payer, hospital location), tobacco use,

Model 2: Model 1 and clinical comorbidities index.

**Sensitivity analysis – hospital cost analysis with missing values imputed**

Table 6: Hospital costs by PTSD

|  | **Women without PTSD** | **Women with PTSD** |
| --- | --- | --- |
| **Cost (US dollars)** |  |  |
| Mean (SD) | 5,692 (4,960) | 7,410 (8,371) |
| Median (IQR) | 4,651 (3,298–6,702) | 5,732 (3,994–8,576) |

Table 7: Adjusted cost ratios among delivery hospitalisations in women with PTSD

| Model | Cost ratio (95% CI) | P value^*^ | Adjusted mean cost, USD (95% CI) |
| --- | --- | --- | --- |
| Unadjusted | 1.30 (1.27 – 1.33) | <0.0001 | 7,410 (7,229 – 7,590) |
| Model 1 | 1.25 (1.22 – 1.27) | <0.0001 | 7,084 (6,931 – 7,236) |
| Model 2 | 1.06 (1.04 – 1.08) | <0.0001 | 6,031 (5,913 – 6,149) |

Model 1: adjusted for sociodemographic characteristics (age group, race and ethnicity, income), hospital factors (payer, hospital location, region), tobacco use, year.

Model 2: Model 1 and clinical comorbidities index.

^*^ Generalised linear model (GLM) with a Gamma distribution and a log link function
